# Minimally invasive beat‐by‐beat monitoring of cardiac power in normal hearts and during acute ventricular dysfunction

**DOI:** 10.14814/phy2.12989

**Published:** 2016-10-04

**Authors:** Audun E. Rimehaug, Eirik Skogvoll, Petter Aadahl, Oddveig Lyng, Dag O. Nordhaug, Lasse Løvstakken, Idar Kirkeby‐Garstad

**Affiliations:** ^1^ Department of Anaesthesiology and Intensive careTrondheim University HospitalTrondheimNorway; ^2^ Department of Circulation and Medical ImagingNTNU, Norwegian University of Science and TechnologyTrondheimNorway; ^3^ Circulation research group Trondheim (CIRCUT)TrondheimNorway; ^4^ Unit of Comparative MedicineNTNU, Norwegian University of Science and TechnologyTrondheimNorway; ^5^ Department of Thoracic SurgeryTrondheim University HospitalTrondheimNorway

**Keywords:** Cardiac power, Doppler ultrasound, echocardiography, heart failure, hemodynamic monitoring

## Abstract

Cardiac power, the product of aortic flow and blood pressure, appears to be a fundamental cardiovascular parameter. The simplified version named cardiac power output (CPO), calculated as the product of cardiac output (CO) in L/min and mean arterial pressure (MAP) in mmHg divided by 451, has shown great ability to predict outcome in a broad spectrum of cardiac disease. Beat‐by‐beat evaluation of cardiac power (PWR) therefore appears to be a possibly valuable addition when monitoring circulatory unstable patients, providing parameters of overall cardiovascular function. We have developed a minimally invasive system for cardiac power measurement, and aimed in this study to compare this system to an invasive method (ttPWR). Seven male anesthetized farm pigs were included. A laptop with in‐house software gathered audio from Doppler signals of aortic flow and blood pressure from the patient monitor to continuously calculate and display a minimally invasive cardiac power trace (uPWR). The time integral per cardiac cycle (uPWR‐integral) represents cardiac work, and was compared to the invasive counterpart (ttPWR‐integral). Signals were obtained at baseline, during mechanically manipulated preload and afterload, before and after induced global ischemic left ventricular dysfunction. We found that the uPWR‐integral overestimated compared to the ttPWR‐integral by about 10% (*P* < 0.001) in both normal hearts and during ventricular dysfunction. Bland–Altman limits of agreement were at +0.060 and −0.054 J, without increasing spread over the range. In conclusion we find that the minimally invasive system follows its invasive counterpart, and is ready for clinical research of cardiac power parameters.

## Introduction

Cardiac power output (CPO) (Bergel et al. 1969), calculated as the product of cardiac output (CO) in L/min and mean arterial pressure (MAP) in mmHg divided by 451, has shown great ability to predict outcome in a broad spectrum of cardiac disease (Williams et al. 2001; Cohen‐Solal et al. 2002; Fincke et al. 2004; Mendoza et al. 2007), outperforming all comparable hemodynamic parameters. CPO can also be combined with systemic vascular resistance to acquire a more precise hemodynamic diagnosis in heart failure, differentiating between septic shock, congestive heart failure, pulmonary edema, hypertensive crisis, and cardiogenic shock (Cotter et al. 2003a,b). Cardiac power (PWR) is the continuous equivalent of CPO, calculated as the product of pressure and flow in the proximal aorta, representing the hydraulic energy transferred from the heart to the vasculature per time unit, excluding the negligible kinetic energy of the blood (Nichols et al. 1977; Boron and Boulpaep 2009; Carlsson et al. 2012). We suggest measuring PWR could improve hemodynamic diagnostics, and that optimizing PWR or PWR‐derived parameters may improve ventriculoarterial coupling (De Tombe et al. 1993; Borlaug and Kass 2009) and outcome in hemodynamically unstable patients. To aid such optimization of cardiac power and research of such applications, we believe the possibility to evaluate PWR live on a beat‐to‐beat basis would be helpful. We have developed a laptop‐based minimally invasive system with this capability, calculating cardiac power based on audio signals (Herr et al. 2010) from Doppler ultrasound of aortic flow and blood pressure from the patient monitor. To validate this minimally invasive system named ultrasound cardiac power (uPWR), we wish to compare it to a previously validated invasive version, transit time cardiac power (ttPWR) (Rimehaug et al. 2013).These systems may perform differently under different physiological and pathophysiological conditions; such as varying degrees of preload/afterload and ventricular dysfunction, motivating this validation study.

The primary aim of this study was to compare our minimally invasive system for cardiac power (uPWR) measurements against its invasive counterpart (ttPWR) under different conditions. The secondary aim was to assess if uPWR measurements may be of help in guiding fluid resuscitation in acute ventricular dysfunction.

## Method

Seven male Noroc pigs (a hybrid of ¼ Duroc, ¼ Yorkshire, and ½ Norwegian landraces) weighing 25–30 kg were included. The protocol was approved by the local steering committee of the Norwegian Experimental Animal Board, “Forsøksdyrutvalgets tilsyns‐ og søknadssystem”, application id 3671. All animals received humane care in compliance with the European Convention on Animal Care (Directive 2010/63/EU). The animal model in use is well established at Trondheim University Hospital, and was adjusted for this study as described below.

### Anesthesia and medical preparations

The animals were premedicated with 4 mg/kg azaperone and 20 mg/kg ketamine given i.m. Prior to operation, the pigs were cleaned and weighed. Anesthesia was then induced using 0.04 mg/kg fentanyl, 10 mg/kg ketamine, 10 mg/kg pentobarbital, and 1 mg of atropine. Respiratory control was achieved by mechanical ventilation through a tracheostomy tube. The ventilator was set in volume‐controlled mode with FiO_2_ = 0.6. The tidal volume was adjusted to obtain normocapnia and a pO_2_ of ≥12 kPa. Anesthesia was maintained using fentanyl 0.02 mg/kg/h and midazolam 0.3 mg/kg/h, and the infusion rate adjusted according to the clinical signs of anesthesia depth. Intravascular volume was maintained through infusion of acetated Ringer's solution and polyhydroxymethylstarch. Boluses of 50 mL of Ringer's solution were added when indicated by central venous pressure (CVP), heart rate, and systemic blood pressure. A bolus of 150 mg of amiodarone was given i.v. to prevent arrhythmia. Hexamethonium at 20 mg/kg was given i.v. to prevent hemodynamic reflex changes during surgical interventions. Isoflurane vapor was given when needed during shorter periods, however, not during or shortly before recording of signals. At the end of the experiment, the animal was euthanized while still under general anesthesia, using 40 mL of pentobarbital 100 mg/mL.

### Surgical preparation

Central venous catheters were inserted in the left jugular vein for infusions and in the right jugular vein for measurement of CVP. Urine production was monitored through cystostomy and bladder catheterization. A catheter was inserted in the right brachial artery for continuous blood pressure monitoring and blood gas sampling. After sternotomy a transit time flow probe was mounted on the ascending aorta, and a micromanometer catheter was inserted into the descending aorta via the left carotid artery. A rubber band was placed around the inferior caval vein for preload reduction, and a balloon catheter was inserted in the ascending aorta via the right femoral artery for afterload increase. A fluid‐filled pressure catheter was placed in the left femoral artery to verify complete occlusion by the balloon catheter. For antithrombotic prophylaxis, 5000 IU of heparin was injected i.v.

### Measurements and calculations

In‐house custom made software (Labview; National Instruments, TX) was used to instantaneously record aortic blood pressure (ABP) from a Millar catheter connected to a CPU‐2000 unit (Millar, Houston, TX), aortic flow and cardiac output from a CardioMed CM4000 transit time flow probe (Medistim, Oslo, Norway), ECG, and brachial artery blood pressure from an Ohmeda Aisys Datex (GE Healthcare, Madison, WI), and Doppler audio signals from aortic flow measured using a GE Vingmed Vivid 7 with a 6T‐probe (GE Vingmed, Horten, Norway). The signals were synchronized in the in‐house software using ECG‐signals from the different apparatus.

Echocardiographic flow data were obtained by placing a GE 6T probe directly on the apex of the heart, achieving a Doppler angle of insonation of 0 degrees. Due to technical limitations with the prototype of our system, we could not record aortic flow in the left ventricular outflow tract immediately below the aortic annulus as planned. Instead the recording was made at the level of the sinotubular junction. We aimed to measure in the center of the cross section, although minor deviations here should have little effect since the flow profile in the aorta can be assumed relatively flat (Ku 1997). Measuring aortic diameter is a known vulnerability, so the diameter was estimated by calibrating ultrasound acquired stroke volume against transit time probe measured stroke volume in 10 consecutive cardiac cycles during baseline conditions before the experiment, by the equation

$$d=\sqrt{\frac{4*\text{ttSV}}{\pi *\text{VTI}}}$$

where ttSV is stroke volume evaluated by the transit time probe, and VTI is the velocity time integral from the Vivid 7 ultrasound scanner. The average diameter calculated from these ten cardiac cycles was then used subsequently as the diameter for the ultrasound Doppler measurements throughout the experiment. The diameter estimate was repeated when the probe was repositioned after the procedure to induce acute ventricular dysfunction. This approach was chosen since estimating the accuracy of measuring piglet aortas was not in the scope of this study, and it allowed us to focus on other possible sources of disagreement between ttPWR and uPWR. The accuracy of measuring the diameter of the human left ventricular outflow tract is well known (Shiran et al. 2009).

**Figure 1:**
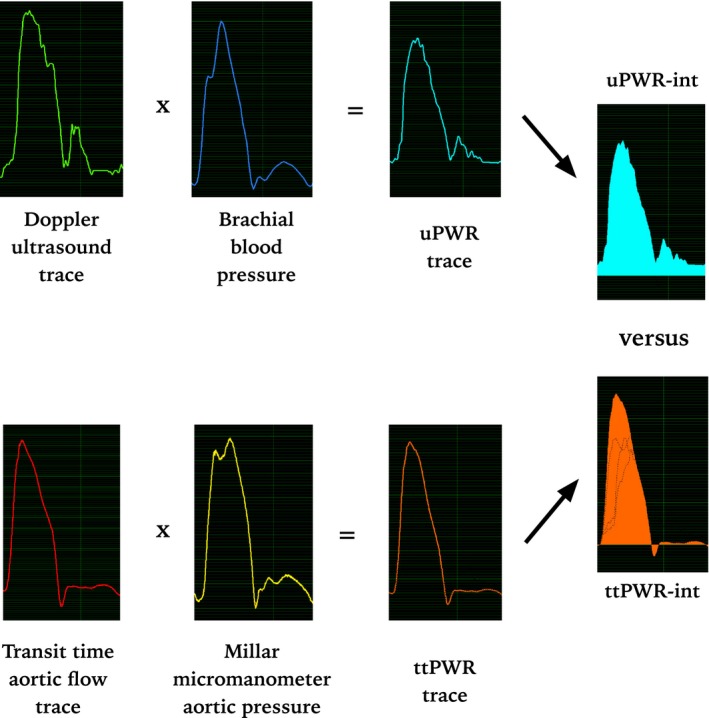
Recordings from in‐house software connected to a GE Vivid 7 ultrasound scanner and an Aisys patient monitor. Brachial blood pressure from a fluid‐filled catheter was exported from the patient monitor, and aortic flow from a GE Vivid 7 to construct live uPWR‐curves (displayed in Watts) using the in house software. Aortic blood pressure from a micromanometer and aortic flow from a transit time probe was simultaneously imported to construct equivalent ttPWR‐curves. The time integral of the uPWR‐curve and the ttPWR‐curve per cardiac cycle, was calculated (in Joules) for comparison of the two curves.

The in‐house developed software was used to calculate two different PWR‐integrals: the uPWR‐integral, based on the aortic flow measured by the Doppler signal combined with the pressure signal from the fluid‐filled catheter in the brachial artery, and the ttPWR‐integral, based on flow measured by the transit time flow probe combined with the pressure determined by the Millar micromanometer in the descending aorta (Rimehaug et al. 2013). This setup is illustrated in Figure 1. The calculation of the PWR‐integrals was performed in the Labview Numeric Integration block IV using the trapezoidal rule:

$$\int_{0}^{T}\text{PWR}\left(t\right)dt\approx \frac{1}{2*f}\sum_{k=1}^{T*f}\left(\text{PWR}\left(t_{k=1}\right)+\text{PWR}\left(t_{k}\right)\right)$$

where *T* is the duration of a given cardiac cycle and f is the sampling frequency, in our case 1000 Hz.

To test the system under different conditions, both preload and afterload was mechanically manipulated before and after inducing acute ventricular dysfunction. Reduced preload was simulated by tightening the rubber band around the inferior caval vein until cardiac output (CO) was reduced by approximately 30%. Increased afterload was simulated by inserting 1–2 mL of water into the balloon catheter placed in the descending aorta until full occlusion was achieved, as determined by a pressure catheter placed in the left femoral artery.

**Figure 2:**
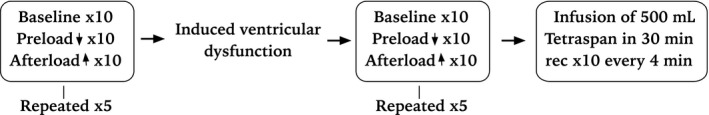
Illustration of the time line of the experiment. Ten cardiac cycles was recorded and averaged for each of the conditions “Baseline”, “Preload reduced”, and “Afterload increased”, this was repeated five times. Ventricular dysfunction was then induced, and the same measurements were thereafter repeated. Finally the infusion part of the experiment was carried out, where ten cardiac cycles was recorded every 4 min.

One set of ten cardiac cycles each was recorded during the three conditions baseline, reduced preload, and increased afterload. Between each recording, the animal was allowed to stabilize. This process was repeated five times, attempting a total of 5*3*10 = 150 recorded cardiac cycles per animal before induction of acute ventricular dysfunction. These measurements were repeated after induced ventricular dysfunction. The entire time line of the experiment is illustrated in Figure 2.

Acute ventricular dysfunction was induced through disseminated microvascular infarction of the left ventricle by injecting 55‐μm polystyrene microspheres in a 500 mg/L concentration in the ostium of the left coronary artery through a 6F coronary artery catheter. Boluses containing microspheres were injected until a 30% reduction in cardiac output was achieved. After 30 min of stabilization, the measurement series described above was repeated, attempting 150 cardiac cycles per animal as above.

### Statistical methods

All signals were simultaneously recorded by in‐house software, from which the results were later exported to a Microsoft Excel spreadsheet prior to import into SPSS (IBM Corp. Released 2011. IBM SPSS Statistics for Windows, Version 20.0; Armonk, NY: IBM Corp.) for statistical analysis and construction of Bland–Altman and regression plots. The relation between the uPWR‐integral and the ttPWR‐integral was first investigated by calculating the overall slope in a linear mixed model with the uPWR‐integral as the dependent and the ttPWR‐integral as the independent variable without intercept and with ventricular dysfunction as a fixed factor, allowing for random slope in each animal. The group indicator (Animal) was included in the analysis by adding interaction between Animal and ttPWR as a random effect.

While the inclusion of random slopes was not significant (*P* = 0.107), we still estimated individual slopes in each animal using a linear model specified as above, acknowledging the low number of animals available for variance estimation. Agreement between slope adjusted uPWR and ttPWR was then investigated using a Bland–Altman plot of differences versus the mean, to investigate if the spread of the difference between uPWR‐integral and ttPWR‐integral tended to increase with increasing values.

The second part of the study was performed after induced acute ventricular dysfunction, and after having obtained all preload‐ and afterload manipulations. An infusion of 500 mL Tetraspan was administered as a constant rate infusion over 30 min, without any other interventions. The uPWR‐integral was recorded for 10 cycles every 4 min, and the average of these ten cycles plotted against time to follow the development. No further statistical analysis was performed on this part of the study, as it was intended only as a feasibility study for tracking effects of volume resuscitation. We instead chose to judge whether uPWR measurements may be of help in guiding fluid resuscitation from the plot of the uPWR‐integral against time/infused volume by the following criteria:

1. An initial positive correlation between fluid infusion and the uPWR‐integral.
2. A clear plateau, defining maximal volume effect.
3. A possible negative correlation between fluid infusion and uPWR‐integral at the end of infusion defining volume overload.

Neither the ventricular dysfunction induced with the microsphere technique nor the volume resuscitation performed during phase one of the experiment are strictly uniform, hence all phases may not be seen in all animals.

## Results

### uPWR‐integral compared to ttPWR‐integral

**Figure 3:**
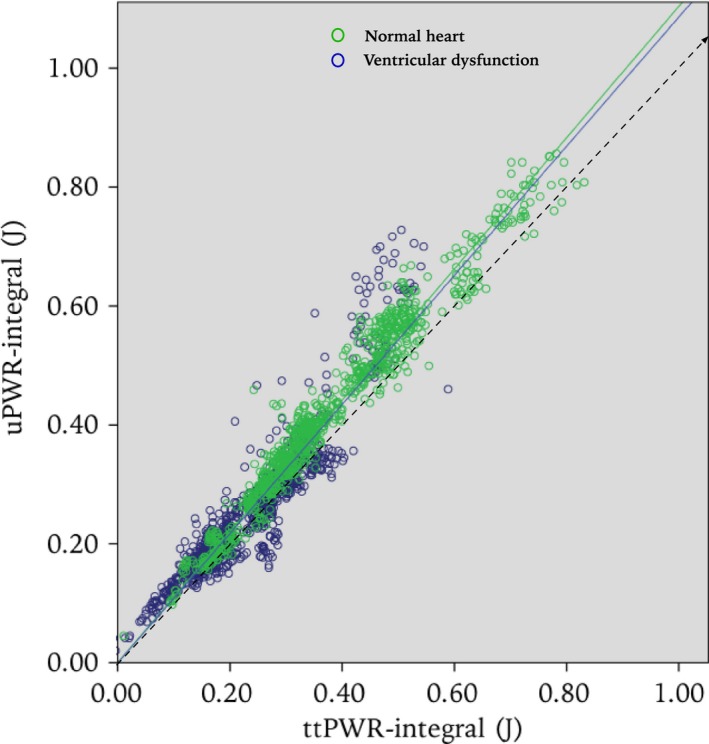
Scatter plots of the uPWR‐integral versus the ttPWR‐integral. Each marker represents one cardiac cycle. Green and blue markers represent before and after induced ventricular dysfunction, respectively, regression lines are added for observations before and after ventricular dysfunction separately. The uPWR‐integral slightly overestimates the ttPWR‐integral by a factor of approximately 1.1, but the relationship between them is seemingly linear

A scatter plot between the uPWR‐integral (uPWR_int_) and the ttPWR‐integral (ttPWR_int_) is given in Figure 3. The linear mixed model analysis showed that uPWR slightly overestimated ttPWR by about 10%, and gave an estimated model with uPWR_int_ = 1.098 * ttPWR_int_ (95% CI 1.068, 1.128, *P*‐value <0.001) for the total material of 1882 observations. When the material was separated into normal heart observations and acute ventricular dysfunction observations, we found the relation uPWR_int_ = 1.104 * ttPWR_int_ (95% CI 1.075, 1.133, *P*‐value <0.001) in normal hearts (962 observations), and the relation uPWR_int_ = 1.086 * ttPWR_int_ (95% CI 1.045, 1.126, *P*‐value <0.001) after acute ventricular dysfunction (920 observations; *P* = 0.001).

For the entire material the difference in the slope between the animals as a random effect was not significant (*P* = 0.107), although the slopes varied in the range 1.06–1.14, meaning that the uPWR measurements overestimated ttPWR by 6–14% in the different animals.

**Figure 4:**
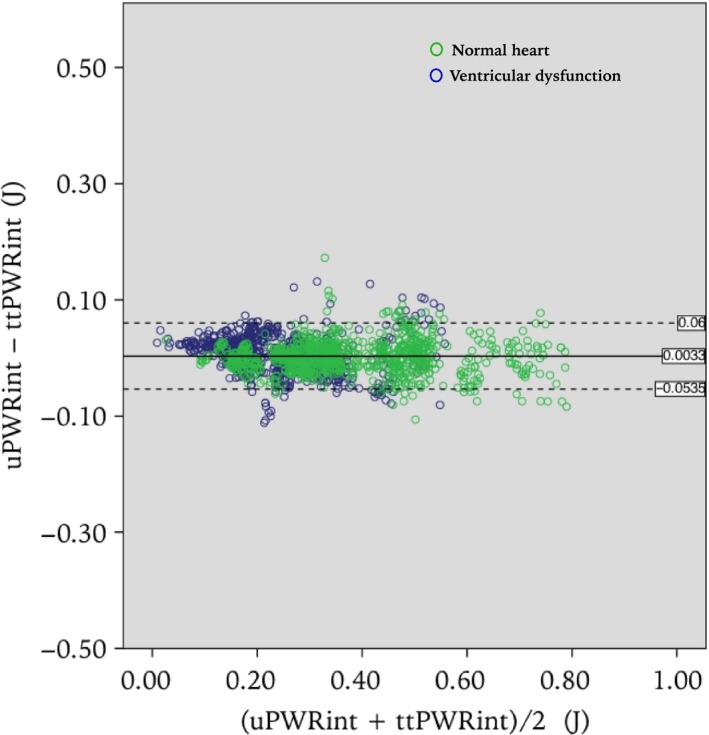
Bland–Altman limits of agreement plots of the uPWR‐integral versus the ttPWR‐integral. The difference between the ttPWR‐integral and the uPWR‐integral is plotted against the average of the two measures. Each marker represents one cardiac cycle. Green and blue markers represent before and after induced ventricular dysfunction, respectively. The limits of agreement have been added for reference, 0.060 and at −0.0535.

A Bland–Altman plot of the difference between uPWR‐integral and the ttPWR‐integral versus their mean is given in Figure 4, with limits of agreement at 0.060 and at −0.0535 J. The plot does not indicate any tendency toward increasing spread of the difference with increasing mean of the uPWR‐integral and ttPWR‐integral.

### uPWR‐integral during fluid resuscitation

**Figure 5:**
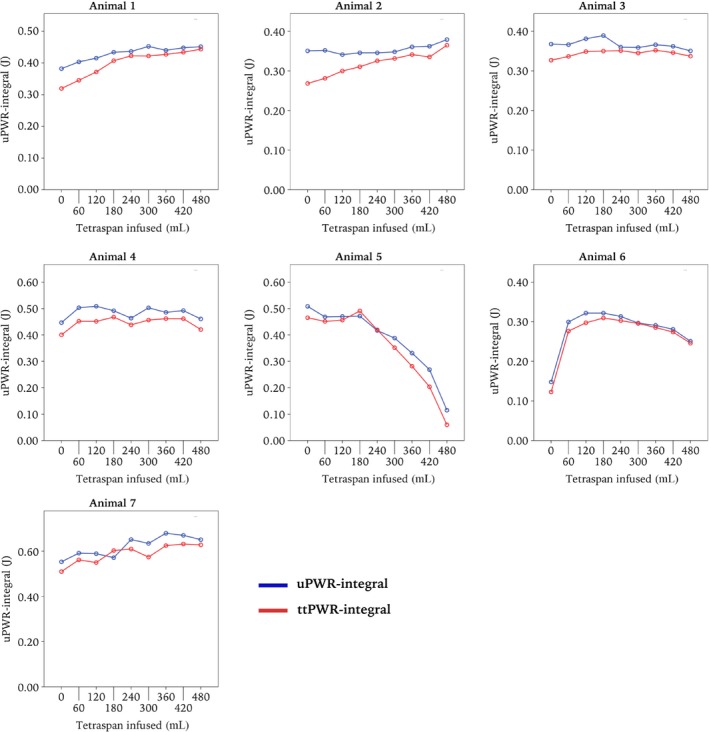
The second part of the study, fluid resuscitation monitored by the uPWR‐integral, with the ttPWR‐integral as a comparison. With manifest ventricular dysfunction, each animal was fluid resuscitated with 500 mL of Tetraspan over 30 min, to illustrate a potential application of the uPWR‐integral in hemodynamic monitoring. We suggest the evaluation of the uPWR‐integral may be able to guide fluid therapy.

The plot of the uPWR‐integral against infusion time/volume differed between animals (Fig. 5). Evaluated by the uPWR‐integral, only animal 1 and 2 benefitted from fluid throughout the entire course, whereas animal 5 and 6 eventually showed detrimental effect of fluid resuscitation. It can also be observed that the uPWR‐integral closely follows the ttPWR‐integral in all animals also in this part of the study.

## Discussion

The minimally invasive uPWR follows the invasive ttPWR when the integrals per cardiac cycle are compared using scatter plot and Bland–Altman limits of agreement. The uPWR‐values from our system need to be divided by approximately 1.1 to match the ttPWR‐measurements. In the second part of our study, our system was able to monitor the uPWR‐integral during fluid resuscitation of hearts with ventricular dysfunction, and seemed to provide information regarding how the individual animal handled the treatment.

From the scatter plot it is apparent that the uPWR‐integral follows changes in the ttPWR‐integral, and the Bland–Altman plot demonstrates that there is no tendency toward increasing spread of the difference between ttPWR and uPWR with increasing values. The relationship between the ttPWR‐integral and the uPWR‐integral is statistically different before and after acute ventricular dysfunction; however, as the difference is of such small value we do not find it biologically nor clinically significant. Regarding absolute values, uPWR will in human studies be vulnerable to error in the measurement of aortic diameter, to the same degree as when considering cardiac output by echocardiography (Otto et al. 1988; Maslow et al. 1996; Valtier et al. 1998; Shiran et al. 2009), a widely accepted technique in clinical use today. Regardless of this vulnerability uPWR‐measurements may well serve to follow a trend, evaluating development and clinical response to treatment.

Part two of this study was performed with the intention to assess if uPWR measurements can be of help in guiding fluid resuscitation in acute ventricular dysfunction. Figure 5 shows the several different courses observed, which illustrate how differently individuals respond to fluid resuscitation. A particular development is seen in animal 6 and 7, where the uPWR‐integral reaches a plateau, before a marked reduction and circulatory failure develops with continued infusion of Tetraspan. We ascribe the interindividual differences as a response to two factors. First; the microsphere method does not give a uniform ventricular dysfunction. Second; this test was performed at the end of a lengthy experiment, during which the animals may have developed different degrees of cardiovascular dysfunctions. Usually considered a methodological weakness, in this case the heterogeneity illustrates the clinical reality. As these piglets are far more homogeneous than patients, the result underlines the need for individualized circulatory treatment in clinical practice. Looking at all the plots in Figure 5 together we consider the criteria for the second aim of the study met.

### Possible applications of uPWR for further research and clinical use

A minimally invasive system for acquiring cardiac power opens several possible applications for further research. As indicated by our feasibility study; by following the uPWR‐integral over time, one could be able to find the limit of tolerated preload in each patient, allowing them to avoid pulmonary edema and other serious complications from excessive fluid resuscitation.

Furthermore, since the PWR‐integral is equivalent to stroke work (Rimehaug et al. 2013) a suggestion is that it can be used to guide treatment, based on the theory that optimal stroke work is a result of optimized ventriculo‐arterial coupling (Borlaug and Kass 2009). In healthy individuals this coupling is optimized physiologically (De Tombe et al. 1993), whereas individuals with acute ventricular dysfunction can be considered to have a coupling problem (Borlaug and Kass 2011). A strategy to optimize the uPWR‐integral should by this reasoning also optimize ventriculo‐arterial coupling. By monitoring blood pressure and flow separately, it can be difficult to evaluate if an intervention has improved or worsened cardiovascular performance, such as when treating systolic heart failure (Borlaug and Kass 2009) with a vasodilator.

Cardiac power output is already available for anyone with basic echocardiographic skills and a blood pressure measurement. We do, however, believe other power parameters may be more useful. CPO represents the *mean* energy delivery per time unit from the heart to the aorta, whereas the uPWR‐integral represents the *total* energy delivery (Westerhof et al. 2010) per heart contraction. The difference between mean and total energy delivery has been shown to be approximately 15% in the left ventricle (Westerhof et al. 2010) in normal conditions, but higher in hypertension (O'Rourke 1967; Nichols et al. 1986). By calculating the uPWR‐integral and comparing to CPO, one would get information about mean, oscillatory, and total energy. Regardless of whether the oscillatory energy is useful or useless (Westerhof et al. 2010), it may be of interest to quantify in the individual patient.

Another possible advantage of uPWR over CPO is that monitoring cardiac power on a beat‐to‐beat basis visualizes respiratory variations. Theoretically cardiac power respiratory variation should be more sensitive to hypovolemia than respiratory variation in flow and pressure (Marik et al. 2011), as power reflects changes in both flow and pressure.

### Limitations of the study

We used brachial artery pressure to calculate uPWR. In our animal model, the pressure measurements from the brachial fluid‐filled catheter and the aortic micromanometer were practically identical. The pigs were young and healthy and had a short distance from the aorta to the brachial artery. In elderly humans with vascular disease, radial pressure does not represent aortic pressure equally well (Pauca et al. 1992). Radial pressure is the usual method for clinical blood pressure measurements, however, arguing for a power curve based on radial pressure as sufficient. An automated adjustment of the time delay between peripheral pressure and aortic flow will be necessary to calculate an instantaneous power curve. Transfer functions from radial to aortic pressure have been explored (Hope et al. 2003; Smulyan et al. 2003; Westerhof et al. 2008, 2010), and could be considered to implement in further development of technology to measure uPWR.

Doppler signals from the ascending aorta were in this study obtained by placing the GE 6T probe directly on the apex of the heart, as we had difficulties obtaining satisfactory images from a transesophageal position in the pigs. In patients, the intention is to obtain these images transesophageally or transthoracally, which are established methods in humans (Poelaert et al. 1999; De Backer 2014).

We calibrated the diameter of uPWR using ttPWR in this study, to avoid disturbances of measurement error of the diameter in the comparison of the two. Such a calibration would not be necessary in human use of uPWR, as the accuracy of measurement of human left ventricular outflow tract is known (Shiran et al. 2009).

The number of included study objects is relatively low, however, with no tendency toward increasing spread in the Bland–Altman plot in any of the animals, and narrow confidence intervals in the relation between the uPWR‐integral and ttPWR‐integral we find that further validation of the low invasive method of uPWR can continue in patients, comparing it to other methods such as CPO calculated by radial blood pressure combined with pulmonary artery catheter measurements.

As both uPWR and ttPWR could be assumed to have a degree of measurement error, investigating the relation should ideally be based on regression with error in both variables (Deming 1943). However, the variance of ttPWR‐measurements are substantially lower than the variance of uPWR‐measurements, making a linear model appropriate (Lundell et al. 1993; Hartman et al. 1994; Bajorat et al. 2006).

## Conclusions

The minimally invasive uPWR‐curve, acquired by ultrasound Doppler audio signals and a brachial artery catheter, provides similar results to the highly invasive ttPWR‐curve based on aortic pressure and flow. The uPWR‐integral seemed able to track cardiovascular performance during fluid therapy. A minimally invasive system for acquiring continuous cardiac power measurements opens several possibilities for research and clinical use of cardiac power parameters.

## Conflict of Interest

The Department of Circulation and Medical Imaging, Norwegian University of Science and Technology has an ongoing cooperation with GE Healthcare (Vingmed Ultrasound).
